# *RASSF1A*基因启动子甲基化与非小细胞肺癌关系的*meta*分析

**DOI:** 10.3779/j.issn.1009-3419.2015.07.09

**Published:** 2015-07-20

**Authors:** 慧君 魏, 念珍 房, 丽丽 郭, 志浩 吴, 清华 周

**Affiliations:** 300052 天津，天津医科大学总医院，天津市肺癌研究所，天津市肺癌转移与肿瘤微环境重点实验室 Tianjin Key Laboratory of Lung Cancer Metastasis and Tumor Microenvironment, Tianjin Lung Cancer Institute, Tianjin Medical University General Hospital, 300052 Tianjin, China

**Keywords:** 肺肿瘤, *RASSF1A*基因, 甲基化, *Meta*分析, Lung neoplasms, *RASSF1A* gene, Methylation, *Meta*-analysis

## Abstract

**背景与目的:**

肿瘤发生、发展过程中，抑癌基因启动子区域CpG岛异常甲基化起着重要的作用。已有研究显示RAS相关区域家族1A(Ras association domain family 1A, *RASSF1A*)基因，作为一个抑癌基因其启动子区域甲基化与非小细胞肺癌(non-small cell lung cancer, NSCLC)的发生发展密切相关。在NSCLC患者癌组织中*RASSF1A*基因启动子往往表现为异常高甲基化。本研究采用*meta*分析的方法探讨*RASSF1A*基因启动子甲基化与NSCLC发生之间的关系。

**方法:**

通过检索Medline、EMBASE、CNKI和万方数据库，按照已拟定的纳入与剔除标准筛选收集公开发表的关于*RASSF1A*基因启动子甲基化与NSCLC相关性的研究。以比值比(odds ratio, OR)和95%置信区间(confidence interval, CI)为效应指标，分析*RASSF1A*基因启动子甲基化与NSCLC的关系。

**结果:**

共有23篇文献纳入本研究，*RASSF1A*基因启动子甲基化率在NSCLC患者肺部组织和对照组中分别为41.50%(95%CI: 34%-49%)和5.58%(95%CI: 2%-9%)，*meta*分析显示肿瘤组织中的甲基化率高于对照组(OR=8.72, 95%CI:4.88-15.58, *P* < 0.05)；亚组分析显示:肿瘤组织中的甲基化频率高于血浆(OR=10.99, 95%CI:2.48-48.68)和正常对照组织(OR=8.74, 95%CI: 4.39-17.41)。

**结论:**

NSCLC患者肺癌组织中*RASSF1A*基因启动子甲基化率高于对照组，组织中*RASSF1A*基因启动子甲基化率对肺癌的发生更具影响，RASSSF1A甲基化可能与肺癌的发生存在相关并可作为肺癌诊断的潜在标志物。

## Abstract (en)

**Background and objective:**

The CpG island aberrant promoter methylation in the tumor suppressor gene region plays an important role in the process of tumorigenesis. Relevant evidence shows that the promoter methylation of RAS association domain family 1A (*RASSF1A*) gene, a tumor suppressor gene, has a close relationship with non-small cell lung cancer (NSCLC) development; therefore, RASSF1A may be a potential NSCLC biomarker. This paper discussed and summarized the relationship between *RASSF1A* gene promoter methylation frequency and NSCLC through *meta*-analysis.

**Methods:**

By searching Medline, EMBASE, CNKI, and Wanfang database, we selected and collected the published articles regarding *RASSF1A* gene promoter methylation and NSCLC risk according to the marked inclusion and exclusion criteria. Through *meta*-analysis, combined odds ratio (OR) and 95% confidence interval (CI) data were used to analyze the *RASSF1A* gene promoter methylation and NSCLC relationship.

**Results:**

A total of 23 articles were utilized in this study. Results indicated that the *RASSF1A* gene promoter methylation rate was 41.50% (95%CI: 34%-49%) in NSCLC tissue and was 5.58% (95%CI: 2%-9%) for the control group. Compared with normal lung tissue, RASSF1A methylation frequency in tumor tissue was significantly higher than that of the control group (OR=8.72, 95%CI: 4.88-15.58, *P* < 0.05). Subgroup analysis showed that the *RASSF1A* gene promoter methylation rate of tumor tissue was higher than that of plasma group (OR=10.99, 95%CI: 2.48-48.68) and normal control tissue group (OR=8.74, 95%CI: 4.39-17.41).

**Conclusion:**

The rate of *RASSF1A* promoter gene methylation in NSCLC patient tissue samples was higher than that of normal lung samples, whereas the rate of *RASSF1A* promoter gene methylation in the tissue has more significant effect on lung cancer occurrence. This finding indicates that *RASSF1A* gene promoter methylation could be used as an NSCLC biomarker and was involved in NSCLC carcinogenic effects.

肺癌是全球范围内发病率和死亡率最高的恶性肿瘤，流行病学数据显示2012年全球肺癌死亡病例约为100万人。非小细胞肺癌(non-small cell lung cancer, NSCLC)是肺癌的主要类型，约占原发性肺癌的80%，其中大约20%的NSCLC适合手术治疗，剩余80%只能接受传统的放化疗^[1]^。近10年来，NSCLC总的5年存活率仅有15.8%^[1]^，其复发转移是治疗失败和患者死亡的主要原因。因此，早期诊断这种疾病是使患者可以长期生存的关键所在。

现有一系列基于分子生物学的方法检测到在肺癌的发生发展过程中，一些肿瘤相关基因发生了异常改变并可以在肿瘤发展的早期阶段检测到，其中相关基因启动子区域的异常甲基化被检测到出现在包括NSCLC在内的多种恶性肿瘤中，并且早于癌症恶性表型出现，可以视为一种早期诊断标志。基因甲基化水平的异常改变已证实是影响基因活性的重要机制之一，可发生在许多肿瘤的早期发生阶段^[2]^。在针对NSCLC患者的研究中，收集相关组织、血液、痰液等进行检测，普遍发现相关抑癌基因(如*RASSF1A*、*MGMT*、*APC*、*E-cadherin*、*P16*等)启动子区域的甲基化发生异常，表明了相关抑癌基因启动子的异常甲基化有可能作为肺癌早期诊断的标志。

近年来，已有多篇研究就抑癌基因甲基化与NSCLC发生的关系之间做了深入探讨，但是有关抑癌基因甲基化率的统计数据波动范围较大，可能是由样本大小、统计方法的差异等原因造成的，为得出相对准确的统计数据，本研究以抑癌基因*RAS*相关区域家族1A(Ras association domain family 1A, RASSF1A)为例，全面搜集了有关*RASSF1A*基因甲基化和NSCLC之间关系的临床研究，利用*meta*分析探讨*RASSF1A*基因启动子甲基化与NSCLC的相关性，为临床的早期诊断提供参考。

## 1 材料与方法

### 1.1 资料来源

检索中英文数据库，包括Medline、EMBASE、CNKI和万方，收集公开发表的关于*RASSF1A*基因启动子甲基化与NSCLC关系的临床研究。检索语种为英语和汉语，分别以“RASSF1A”、“methylation”、“lung cancer”、“lung carcinoma”、“non-small cell lung carcinoma”为主题词和自由词，检索Medline和EMBASE英文数据库；以“RASSF1A”基因、“甲基化”、“肺癌”、“非小细胞肺癌”、“肺肿瘤”为关键词或题名检索CNKI和万方等中文数据库，检索日期截止为2015年3月，同时辅以手工检索收集各种杂志公开发表的学术论文，学位论文和会议记录摘要等。

### 1.2 文献纳入和排除标准

#### 1.2.1 纳入标准

① 原创论文；②研究对象为NSCLC患者且不限制病程分期；③检测*RASSF1A*基因启动子仅采用甲基化特异聚合酶链式反应(methylation specific PCR, MSP)，实时MSP(Real Time-MSP, RT-MSP)及定量MSP(Quantitative -MSP, Q-MSP)方法；④研究结果提供实验组(癌组织)与对照组(正常组织、血清、或支气管灌洗液等)的甲基化；⑤随机选择病例。

#### 1.2.2 排除标准

① 非临床研究型论文；②样本量过少(*n* < 10)；③论文质量较差或者数据不清；④不符合纳入标准的。如有作者以第一作者，同一单位发表多篇相关且内容类似的论文，取最新的研究结果纳入。

### 1.3 文献质量评价

根据“观察性流行病学研究报告规范(STROBE)-病例-对照研究”对所纳入的文献进行了量化评价。

### 1.4 数据提取

对于所纳入的文献进行相关数据提取，包括：①第一作者姓名、研究所在地、标题、期刊名称、发表年份等；②实验组和对照组中*RASSF1A*基因启动子甲基化的发生率。

### 1.5 统计分析

采用STATA/SE 11.0(StataCorp LP, http://www.stata.com)软件进行统计分析，以NSCLC患者癌组织与正常对照组*RASSF1A*基因启动子甲基化率的优势比OR(odds ratio)或者95%置信区间(CI)作为统计指标。首先用*I*^2^进行统计学异质性评估，如果纳入各研究间存在统计学异质性(*I*^2^ > 0.5)，则采用随机效应模型进行分析。如果研究结果之间无异质性则采用固定效应模型^[3]^合并数据。利用灵敏度分析来评估纳入的每项研究对*meta*分析最终结果的作用，*Begg*的漏斗图和*Egger*测试都是用来评估可能的发表偏倚^[4]^，采用*pesrson*等级相关测试对比*RASSF1A*基因启动子甲基化与实验组和对照组的关系。

## 2 结果

### 2.1 文献情况

检索相关数据库，最初检索到相关文献186篇，发表于2001年-2015年，严格依据纳入和排除标准，在阅读标题、摘要及全文后剔除163篇，最终有23篇文献^[5-27]^符合要求并纳入本*meta*分析(图 1)。原始研究均提供了较为完整的数据资料，23篇研究中，18篇在亚太地区进行(13篇在中国，2篇在韩国，3篇在日本)，剩下的2篇在美国，1篇在德国，1篇在新西兰，1篇在瑞士。研究还提供了研究对象的年龄，组织病理学类型，肿瘤阶段，基因甲基化状态等基本资料(表 1)。

**1:**
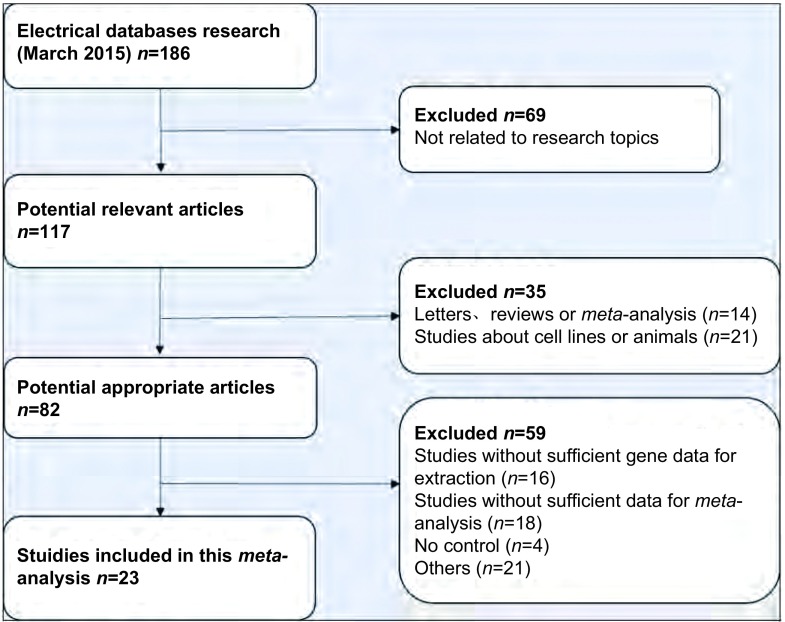
文献筛选流程图 Flowchart of the literature search procedure

**1:** 纳入研究的对象的基本特征 Main characteristics of the included studies

Author	Tumor tissue	Cotrol tissue	Control type	Stage	Method	Region	Time
	(M+/M-)	(M+/M-)		(Ⅰ/Ⅱ/Ⅲ/Ⅳ/other)			
Hubers^[5]^	31/73	3/86	Sputum	14/9/24/25/1	MSP	Netherland	2015
Zhai^[6]^	22/42	0/40	Plasm	4/2/14/22/-	MSP	China	2014
Li^[7]^	48/56	0/56	Plasm	15/7/20/14/-	MSP	China	2014
Tan^[8]^	55/200	34/200	Plasm	-	q-MSP	China	2013
Li^[9]^	45/56	0/52	Plasm	15/7/20/14/-	MSP	China	2012
Lee^[10]^	92/206	1/40	Tissue	130/76/-	MSP	Korea	2012
Song^[11]^	31/78	6/78	Tissue	25/33/19/1/-	MSP	China	2011
Zhang^[12]^	58/150	0/20	Tissue	49/32/48/21/-	MSP	China	2010
Helmbold^[13]^	17/18	0/18	Tissue	-	MSP	Germany	2009
Lin^[14]^	53/124	2/26	Tissue	-	MSP	China	2009
Liu^[15]^	40/60	7/60	Tissue	-	MSP	China	2008
Brock^[16]^	25/50	37/104	Tissue	-	MSP	USA	2008
Yu^[17]^	23/75	0/50	Plasm	9/18/28/20/-	MSP	China	2008
Yanagawa^[18]^	43/101	3/101	Tissue	68/7/26/-/-	MSP	Japan	2007
Wang^[19]^	23/75	0/15	Plasm	-	MSP	China	2007
Hsu HS^[20]^	30/63	3/36	Tissue/Plasm	41/21/1	MSP	China	2007
Chen^[21]^	44/114	4/57	Tissue	50/64/-	MSP	China	2006
Ito^[22]^	44/138	0/138	Tissue	-	MSP	Japan	2005
Fujiwara^[23]^	11/91	8/100	Plasm	53/7/22/9/-	MSP	Japan	2005
Safar^[24]^	19/105	2/32	Tissue	38/8/32/22/-	MSP	USA	2005
Choi^[25]^	47/116	0/116	Tissue	68/23/25/-/-	MSP	Korea	2005
Wang^[26]^	46/119	4/119	Tissue	-	MSP	China	2004
Burbee^[27]^	32/107	0/104	Tissue	60/21/25/-/-	MSP	Sweden	2001
MSP: methylation specific PCR; Q-MSP: Quantitative MSP.							

### 2.2 纳入研究的文献质量评价

根据“观察性流行病学研究报告规范(strengthening the reporting of observational studies in epidemiolog STROBE)-病例-对照研究”进行了量化评价，总体比较，英文文献质量好于中文文献(表 2)。

**2:** 纳入研究的23篇文献的质量评价(STROBE声明) STROBE Statement-checklist criteria of 23 reports included in this study

Items	Recommendation	Number of stydy
Title and abstract	1.1 Indicate the study’s design with a commonly used term in the title or the abstract	23 (100%)
	1.2 Provide in the abstract an informative and balanced summary of what was done and what was found	23 (100%)
Introduction		
Background/Rationale	2 Explain the scientific background and rationale for the investigation being reported	23 (100%)
Objectives	3 State specific objectives, including any prespecified hypotheses	23 (100%)
Methods		
Stydy desin	4 Present key elemens of study design early in the paper	23 (100%)
Setting	5 Describe the setting, locations, and relevant dates, including periods of recruitment, exposure, follow-up, and data collection	23 (100%)
Participants	6.1 Give the eligibility criteria, and the sources and methods of ascertainment, and control selection.Give the rationale for the choice of cases and controls	18 (78.3%)
	6.2 For matched studies, give matching criteria and the number of controls per case	12 (52.2%)
Variables	7 Clearly define all outcomes, exposures, predictors, potential confounders, and effect modifiers. Give diagnostic criteria, if applicable	11 (47.8%)
Datasources/Measurement	8 For each variable of interest, give sources of data and details of methods of assessment (measurement). Describe comparability of assessment methods if there is more than one group	10 (43.5%)
Bias	9 Describe any efforts to address potential sources of bias	3 (13%)
Study size	10 Explain how the study size was arrived at	0 (0)
Quantitative variables	11 Explain how quantitative variables were handled in the analyses. If applicable, describe which groupings were chosen and why	5 (21.7%)
Statistical methods	12.1 Describe all statistical methods, including those used to control for confounding	20 (87%)
	12.2 Describe any methods used to examine subgroups and interactions	12 (52.2%)
	12.3 Explain how missing data were addressed	8 (34.8%)
	12.4 If applicable, explain how matching of cases and controls was addressed	7 (30.4%)
	12.5 Describe any sensitivity analyses	7 (30.4%)
Results		
Participants	13.1 Report numbers of individuals at each stage of study—*eg* numbers potentially eligible, examined for eligibility, confirmed eligible, included in the study, completing follow-up, and analysed	20 (87%)
	13.2 Give reasons for non-participation at each stage	4 (17.4%)
	13.3 Consider use of a flow diagram	0 (0)
Descriptive data	14.1 Give characteristics of study participants (eg demographic, clinical, social) and information on exposures and potential confounders	18 (78.3%)
	14.2 Indicate number of participants with missing data for each variable of interest	5 (21.7%)
Outcome data	15 Report numbers in each exposure category, or summary measures of exposure	15 (65.2%)
	16.1 Give unadjusted estimates and, if applicable, confounder-adjusted estimates and their precision (eg, 95% confidence interval). Make clear which confounders were adjusted for and why they were included	14 (60.9%)
	16.2 Report category boundaries when continuous variables were categorized	17(73.9%)
	16.3 If relevant, consider translating estimates of relative risk into absolute risk for a meaningful time period	3 (13%)
Other analyses	17 Report other analyses done—*eg* analyses of subgroups and interactions, and sensitivity analyses	5 (21.7%)
Discusion		
Key results	18 Summarise key results with reference to study objectives	23 (100%)
Limitations	19 Discuss limitations of the study, taking into account sources of potential bias or imprecision. Discuss both direction and magnitude of any potential bias	14 (60.9%)
Interpretation	20 Give a cautious overall interpretation of results considering objectives, limitations, multiplicity of analyses, results from similar studies, and other relevant evidence	21(91.3%)
Generalisability	21 Discuss the generalisability (external validity) of the study results	19 (82.6%)
Other information		
Funding	22 Give the source of funding and the role of the funders for the present study and, if applicable, for the original study on which the present article is based	15(65.2%)

### 2.3 不同组织甲基化率比较

#### 2.3.1 总体比较

本篇*meta*分析所纳入的研究中经统计NSCLC患者肺癌组织中*RASSF1A*基因启动子甲基化的发生率为41.50%(95%CI: 34%-49%)，对照组肺部正常组织中RASSF1A启动子的甲基化率为5.58%(95%CI: 2%-9%)。两组相比，*RASSF1A*基因启动子甲基化率有统计学差异(*P* < 0.05)(图 2A)。

**2:**
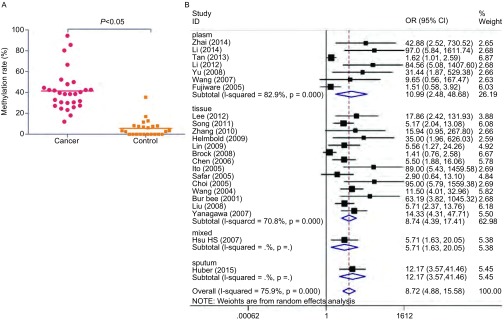
不同组织甲基化率比较。A：肺癌样本与正常样本中RASSF1A基因启动子甲基化率的对比；B：肺癌样本各亚型中*RASSF1A*基因启动子甲基化率的森林图。 Comparison of methylation rate in different tissues. A: Comprison of *RASSF1A* gene promoter methylation between lung cancer samples and normal samples. B: Forest plot of *RASSF1A* gene promoter methylation in subgroup of lung cancer samples.

#### 2.3.2 亚组分析

对纳入的肺癌病例进行组织来源的亚组分析，在7例血浆样本中*RASSF1A*基因启动子甲基化的发生频率低于相应的肿瘤组织(OR=10.99, 95%CI: 2.48-48.68)；在14例正常肺组织样本中*RASSF1A*基因启动子甲基化频率低于相应的肺癌组织(OR=8.74, 95%CI: 4.39-17.41)(图 2B)。

### 2.4

异质性检验以肺癌中组织中*RASSF1A*基因启动子甲基化率的优势比OR为效应量，进行统计学异质性检验。肺癌组织与正常肺组织比较*I*^2^=82.3%，*P* < 0.01，存在统计学异质性(图 3)，因此采用随机效应模型进行分析。

**3:**
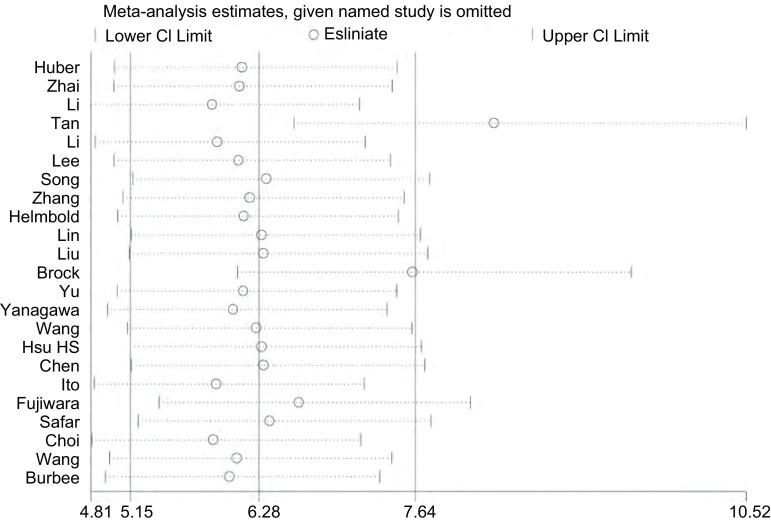
异质性分析 Heterogeneity analyse

### 2.5 敏感性分析

对纳入研究的文献进行敏感性分析，逐一剔除其中的每一篇文献后计算OR值，结果显示逐一剔除每一篇文献后OR值均大于1，*P* < 0.05，说明研究结果较为稳定，对其纳入其中的文献不敏感。

### 2.6 发表偏倚的评估

漏斗图评估发表偏倚(*P* < 0.05)，有8项研究落在了95%CI外，说明该研究存在明显的发表偏倚(图 4)。

**4:**
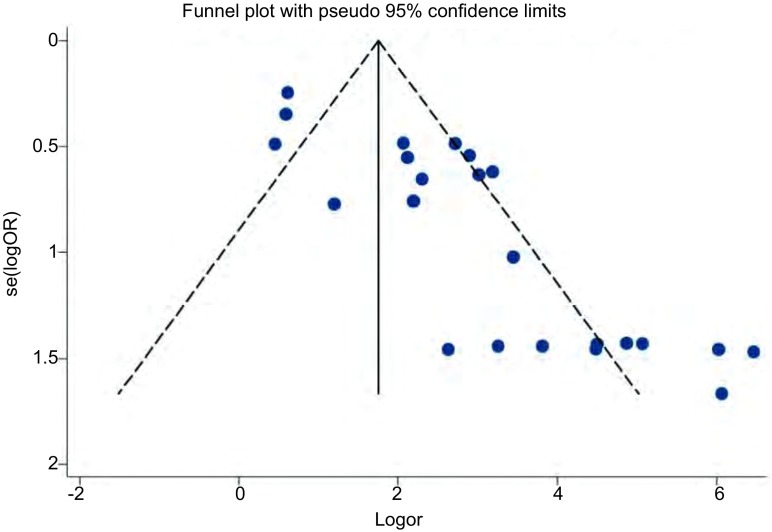
*Begg*漏斗图法评估发表偏倚 Publication bias evaluation by *Begg* funnel plot

## 3 讨论

DNA甲基化是最早发现的基因修饰途径之一，普遍存在于动植物中，研究^[28]^表明DNA甲基化能引起染色质结构、DNA构象、稳定性及其与蛋白质相互作用方式的改变，从而控制基因表达，导致基因突变或者基因沉默等。抑癌基因启动子区域的甲基化异常是基因失活的重要机制之一，可能导致基因转录沉默，影响了细胞的代谢、凋亡等，与肿瘤的发生发展有着密切联系。RAS相关区域家族1A(*RASSF1A*)是由Dammann等^[29]^在2000年发现的抑癌基因，位于染色体3p21.3区，可以通过多种途径抑制肿瘤形成，其启动子区域的甲基化可导致基因失活。在正常组织中*RASFF1A*基因启动子区域很少发生甲基化，其主要发生在肿瘤组织，如肺癌、乳腺癌中^[30]^。目前*RASSF1A*基因启动子区甲基化水平已经成为多种肿瘤诊断的一个重要生物学指标，统计已有文献发现RSSF1A启动子的甲基化率在肺癌患者中为12.1%-94.4%^[13, 23]^，在对照组的标本中为0-35.5%^[17]^，数据范围浮动较大，原因可能是由于各研究中所选样本大小、特征的差异所造成的，因此本研究收集各项有关*RASSF1A*基因启动子甲基化与NSCLC关系的研究，采用*meta*分析的方法系统的整合数据，为临床提供更可靠的证据。

本研究共纳入23篇符合要求的文献，*meta*分析后发现与对照组相比，肿瘤组织中的甲基化率高于对照组(OR=8.72, 95%CI:4.88-15.58, *P* < 0.05)，说明*RASSF1A*启动子高甲基化与肺癌的发生可能存在正相关性，即*RASSF1A*基因启动子甲基化率越高，NSCLC的患病率可能就越高。亚组分析显示肿瘤组织中的甲基化频率高于血浆(OR=10.99, 95%CI: 2.48-48.68)和正常对照组织(OR=8.74, 95%CI: 4.39-17.41)，表明肺癌组织中的甲基化频率对肺癌的发生更具影响。综上，NSCLC中的*RASSF1A*基因启动子的甲基化率可以给未来的临床研究和治疗提供依据。但是由于该*meta*分析的局限性，分析结果需谨慎对待，尚需更多合理的设计进一步证实。

本篇*meta*分析的局限性在于：①研究间存在明显的统计学异质性(*I*2=82.3%, *P* < 0.01)，虽然我们采用了随机效应模型进行分析，但统计学异质性对结果的稳定性产生很大影响。②*Begg*漏斗图显示该研究存在明显的发表偏倚，原因可能是多方面的，譬如一些相关阴性结果未被关注或未被及时发表，各国文献收录之间存在差异等。所以本篇*meta*所得出的结论稳定性可能稍差。③本篇只选用了RASSF1A启动子甲基化率作为唯一变量，研究其与NSCLC之间的关系，但是多篇报道显示在NSCLC中，RASSF1A的高甲基化可能联合多种因素，如年龄、性别、吸烟状况等对癌症的发生发展起到重要的作用，所以在以后的研究中，可以考虑纳入多因素变量进行全面的统计分析。

总的来说，*RASSF1A*基因启动子的甲基化与NSCLC的发生发展是有密切联系的，其作为NSCLC早期诊断的分子标志物有一定的说服力，但是还需要更完善更合理的数据统计和研究设计来为日后的临床治疗提供有力的指导。
